# Merging Plastics, Microbes, and Enzymes: Highlights from an International Workshop

**DOI:** 10.1128/aem.00721-22

**Published:** 2022-06-28

**Authors:** Diego Javier Jiménez, Başak Öztürk, Ren Wei, Timothy D. Bugg, Carol Viviana Amaya Gomez, Felipe Salcedo Galan, Jinneth Lorena Castro-Mayorga, Juan Fernando Saldarriaga, Natalia Andrea Tarazona

**Affiliations:** a Microbiomes and Bioenergy Research Group, Department of Biological Sciences, Universidad de los Andes, Bogotá, Colombia; b Junior Research Group Microbial Biotechnology, Leibniz Institute DSMZ—German Collection of Microorganisms and Cell Cultures, Braunschweig, Germany; c Junior Research Group Plastic Biodegradation, Institute of Biochemistry, University of Greifswald, Greifswald, Germany; d Department of Chemistry, University of Warwick, Coventry, United Kingdom; e Corporación Colombiana de Investigación Agropecuaria (Agrosavia), Mosquera, Colombia; f Department of Chemical and Food Engineering, Universidad de los Andes, Bogotá, Colombia; g Department Civil and Environmental Engineering, Universidad de los Andes, Bogotá, Colombia; h Institute of Active Polymers, Helmholtz-Zentrum Hereon, Teltow, Germany; Shanghai Jiao Tong University

**Keywords:** biobased plastics, biodegradation, enzyme engineering, lignocellulose, microbial evolution, microbial consortia, microbiomes, plastic recycling, polymer science, polyethylene terephthalate

## Abstract

In the Anthropocene, plastic pollution is a worldwide concern that must be tackled from different viewpoints, bringing together different areas of science. Microbial transformation of polymers is a broad-spectrum research topic that has become a keystone in the circular economy of fossil-based and biobased plastics. To have an open discussion about these themes, experts in the synthesis of polymers and biodegradation of lignocellulose and plastics convened within the framework of The Transnational Network for Research and Innovation in Microbial Biodiversity, Enzymes Technology and Polymer Science (MENZYPOL-NET), which was recently created by early-stage scientists from Colombia and Germany. In this context, the international workshop “Microbial Synthesis and Degradation of Polymers: Toward a Sustainable Bioeconomy” was held on 27 September 2021 via Zoom. The workshop was divided into two sections, and questions were raised for discussion with panelists and expert guests. Several key points and relevant perspectives were delivered, mainly related to (i) the microbial evolution driven by plastic pollution; (ii) the relevance of and interplay between polymer structure/composition, enzymatic mechanisms, and assessment methods in plastic biodegradation; (iii) the recycling and valorization of plastic waste; (iv) engineered plastic-degrading enzymes; (v) the impact of (micro)plastics on environmental microbiomes; (vi) the isolation of plastic-degrading (PD) microbes and design of PD microbial consortia; and (vii) the synthesis and applications of biobased plastics. Finally, research priorities from these key points were identified within the microbial, enzyme, and polymer sciences.

## INTRODUCTION

In 2020, around 400 million metric tons (MT) of synthetic plastics was globally produced, and further increases are expected after the coronavirus disease 2019 (COVID-19) pandemic to almost 600 MT per year by 2050 (1). Currently, many ecosystems across the world (e.g., oceans, freshwater, mangroves, coral reef, agricultural soils, forests, polar regions, and the atmosphere) are threatened by plastic pollution. This can negatively affect biosphere wildlife; for instance, marine animals (e.g., birds, mammals, and fish) can swallow plastic debris or become entangled in plastic fishing nets, causing death due to starvation or suffocation (2). In nature, plastic debris can be turned into microplastics (MPs; defined as particles up to 5 mm in dimensions) by physical and chemical erosion. MPs and nanoplastics (NPs; particles <100 nm) could serve as a sink for toxic compounds, increasing their negative impact on terrestrial and marine environments (3). These tiny particles can also be released during the use of plastic products (e.g., fibers from textiles or tire wear) (4, 5) and are widespread on our planet, including having been found in human blood (6). While NPs have not been extensively measured in the environment, there is increasing concern that they may be more hazardous than MPs, mainly because of their potential capability of permeating biological membranes (7).

A large fraction of plastic pollution comes from excessive use of single-use products (e.g., bottles, bags, food containers, cups, and face masks) and the inadequate waste management in urban and rural zones. It is widely reported that more than 90% of the total global plastic production utilizes fossil-based materials, made of polyethylene (PE), polypropylene (PP), polyvinyl chloride (PVC), polyurethanes (PUs), polystyrene (PS), and polyethylene terephthalate (PET) (8). These plastics can persist in nature for tens to thousands of years, depending on the material and environmental conditions, such as the availability of oxygen and sunlight exposure (9). For instance, a plastic bottle made of high-density PE with a wall thickness of ~500 μm will take 250 years to lose the first 50% of the polymer mass (half-life) when buried inland or 58 years to do the same in a marine environment (10). Moreover, the production of these durable plastics can release more than 850 MT/year of CO_2_ into the atmosphere (11). Currently, the production of biodegradable and biobased plastics, for instance, polylactic acid (PLA), polyhydroxyalkanoates (PHAs) and thermoplastic starch (TPS), could be an attractive option to reduce the carbon footprint and for specific applications where a short life span is expected (12). However, their market is still limited, and their biodegradability and recyclability in various end-of-life scenarios need to be carefully evaluated (11). It is important to note that biobased plastics (those derived from biological sources) are not always biodegradable, and some biodegradable plastics are derived from fossil resources (e.g., polybutylene adipate-co-terephthalate [PBAT]). Plastics that are biobased, biodegradable, or both are known as bioplastics. However, the term “bioplastic” is very broad and sometimes is erroneously used, causing misconception among the general public and academists. Accurate definitions of “biobased plastics,” “bioplastics,” “biodegradable plastics,” and “fossil-based plastics” are highly important within this field. Recently, Wei et al. (13) and Rosenboom et al. (11) summarized accurate definitions of these terms in association with distinct polymer types (Table 1).

**TABLE 1:** Terms, definitions, and examples of polymers associated with different types of plastics

Term	Biodegradable polymers^*a*^	Polymers that are nonbiodegradable, resistant to degradation, or durable^*b*^	Definition
Biobased (biorefinery)	PHAs, PLA, polysaccharide-based, plastics (e.g., TPS), bio-polybutylene succinate	Bio-PE, bio-PP, bio-PET, bio-PUs, polyethylene furanoate	Derived from renewable resources (e.g., lignocellulose)
Fossil-based (traditional refinery)	PBAT, polyvinyl alcohol	PET, PP, PE, PS, PVC, PUs	Derived from fossil resources (e.g., oil)

a Polymers with enzymatically accessible backbones that can be cleaved into their respective oligomers/monomers.

b Polymers with saturated C-C backbones or with other chemical bonds highly resistant to biocatalytic depolymerization.

Among other strategies to reduce the negative impacts of fossil-based plastics in the biosphere, the exploration of microbial diversity for plastic transforming activities is a timely and highly relevant topic worldwide (14, 15). Thus, several studies have been carried out to decipher the biofilm-forming microbial communities associated with plastic particles (the so-called “plastisphere”) (16–18). These communities could be different from those found in the environmental matrix (e.g., soil or water) that contains the plastic particles (known as the surrounding plastisphere) (19), probably because of distinct positive/negative interactions, selective pressure, and niche preferences. It has been suggested that some plastics can be depolymerized in natural ecosystems by microbial action. However, a holistic ecoevolutionary and mechanistic understanding of plastic transformation by microbial communities (*Bacteria*, *Archaea*, and *Fungi*), and their enzymes, is still in its infancy (20–22). Moreover, the catabolism of certain biodegradable plastics is supposed to follow principles similar to those of the transformation of plant-derived polymers (e.g., lignocellulose and cutin). For plant biomass and plastic biodegradation, enzymes secreted by microorganisms can catalyze the depolymerization reactions (e.g., by hydrolysis or oxidation of the polymer backbones), releasing (in some cases) monomers or oligomers that are small enough to be translocated to the cytosol, where they can be assimilated and used a carbon source to produce biomass and carbon dioxide or methane (23, 24). In this article, the term “biodegradation” is used as a synonym for modification, depolymerization, and/or mineralization of polymers by microorganisms and their enzymes.

**FIG 1:**
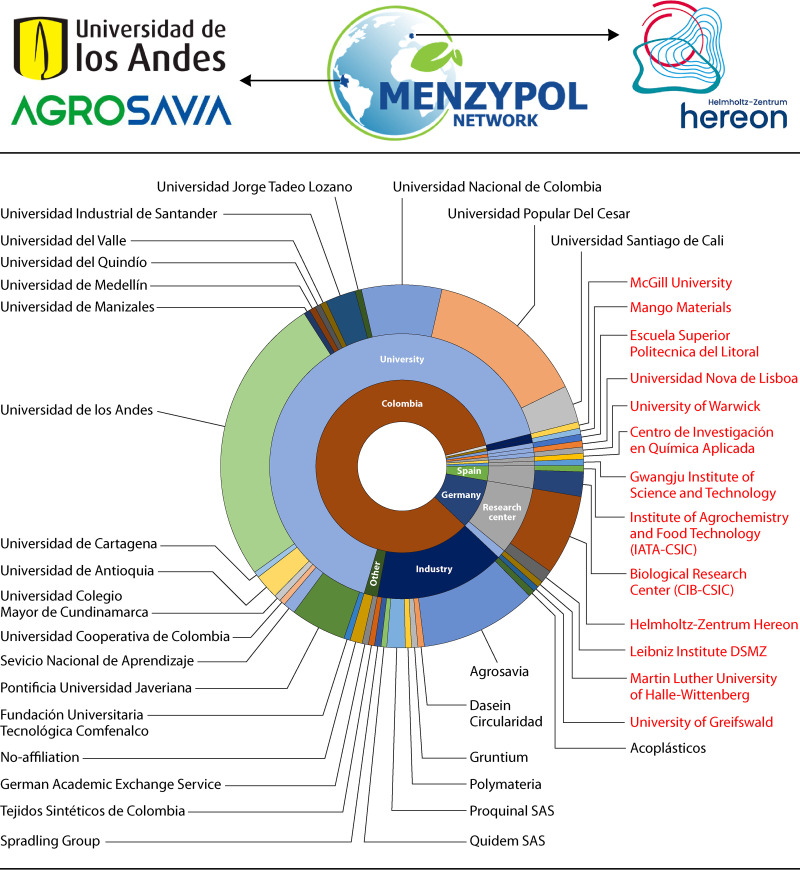
Logos of MENZYPOL-NET partners and pie chart of participants (180 registered persons from industry and academic institutions) in the workshop “Microbial Synthesis and Degradation of Polymers: Toward a Sustainable Bioeconomy.” Names in red are those of non-Colombian institutions.

**FIG 2:**
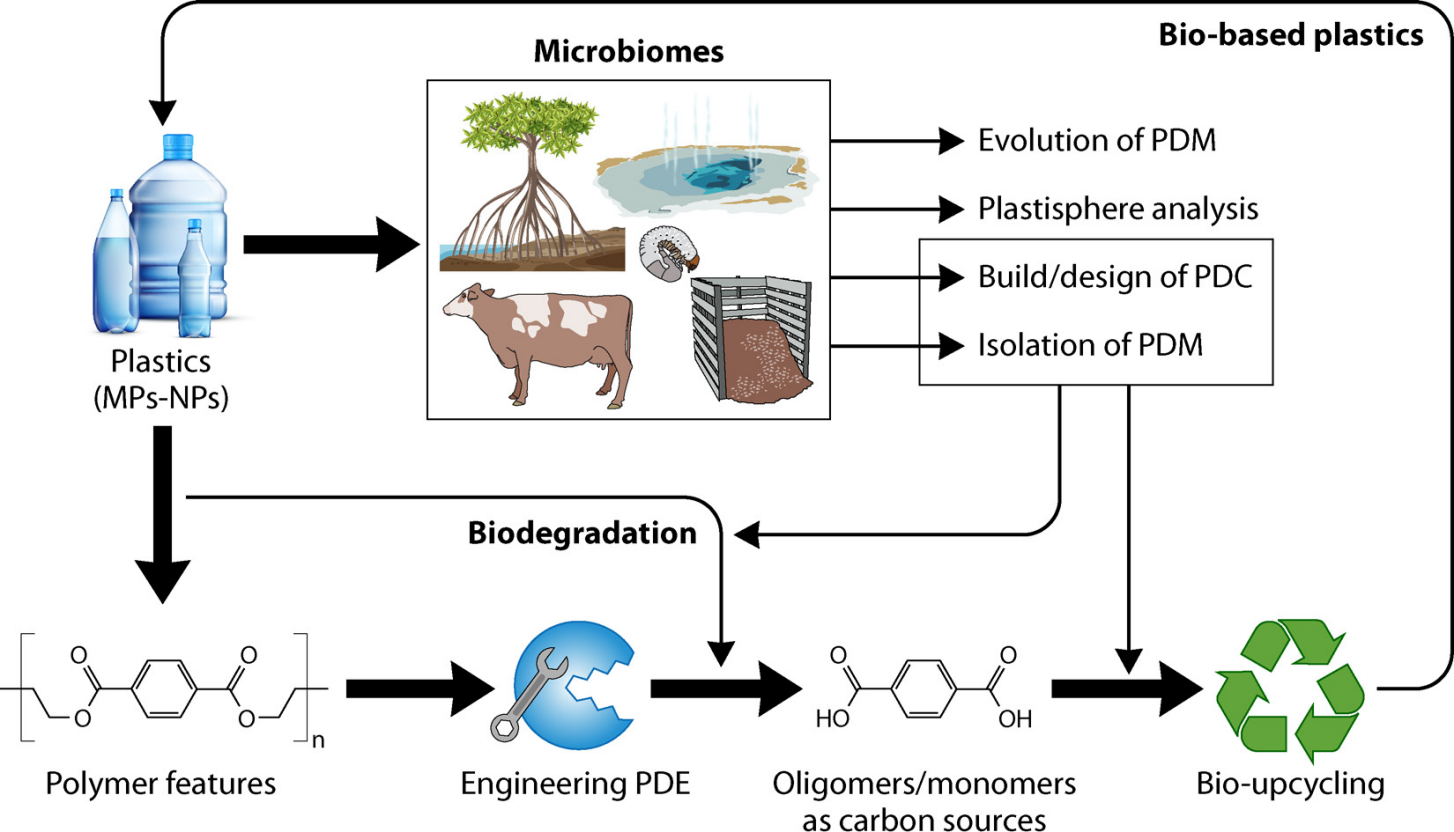
Main topics of discussion, ideas, conclusions and perspectives (and the interplay between them) after the MENZYPOL-NET 2021 workshop (“Microbial Synthesis and Degradation of Polymers: Toward a Sustainable Bioeconomy”). Briefly, the relevance of studying the impact of microplastics (MPs) and nanoplastics (NPs) in different microbiomes (e.g., mangrove soil, agricultural soils, hot springs, compost, and cow rumen) is highlighted. These studies could give new insights into (i) the functional and ecological understanding of the plastisphere and (ii) the microbial evolution driven by plastic pollution. In addition, these ecosystems can be an excellent source of novel plastic-degrading microbes (PDM) or consortia (PDC), which could play key roles in a prospective plastic bioconversion systems (e.g., biodegradation and bio-upcycling of plastic-derived monomers). However, a comprehensive understanding of polymer features (e.g., type of backbones, additives, molecular weight, and hydrophobicity), in addition to efficient engineering of plastic-degrading enzymes (PDE), is required to improve these systems.

The microbial transformation of polymers (e.g., fossil-based plastics, biodegradable plastics and natural polymers) is broadly discussed by the scientific community. However, there are still open questions that must be tackled in an international sphere and by intertwining different branches of science. The Transnational Network for Research and Innovation in Microbial Biodiversity, Enzymes Technology and Polymer Science (MENZYPOL-NET) was created in 2021 by early-career scientists from Helmholtz-Zentrum Hereon (Germany), The Andes University, and The Colombian Corporation of Agricultural Research (Agrosavia) (Colombia) to address challenges in the development of sustainable polymer materials within the scope of a circular bioeconomy. Within the framework of MENZYPOL-NET and its first international workshop (“Microbial Synthesis and Degradation of Polymers: Toward a Sustainable Bioeconomy,” held on 27 September 2021 via Zoom) (Fig. 1), we invited external researchers (a complete list of panelists, guest experts, and organizers is found in Table 2) to have an open discussion in two parallel topic sessions: (i) microbial/enzymatic degradation of plant-derived polymers and plastics and (ii) synthesis and applications of biobased polymers. To deepen these topics, four main questions were brought to the discussion. (i) how can the biodegradation and/or recycling of fossil-based and biobased plastics be improved? (ii) Which strategies can be useful to discover novel microbes/enzymes involved in the biodegradation and/or recycling of durable plastics? (iii) How can we extrapolate the understanding of lignocellulose transformation in plastic biodegradation? (iv) What are the constraints for biobased and biodegradable plastic production, use, commercialization and recycling? From the experts’ input, the following eight key points were highlighted, also depicted in Fig. 2. Based on them, several research priorities were identified to better understand the transformation of durable plastics, including their biodegradation, their recycling, and the consequences of their presence in the biosphere. We have divided them into three main fields: polymer, enzyme, and microbial science (Table 3).

**TABLE 2:** Panelists, guest experts, and organizers of the MENZYPOL-NET workshop 2021

Name	Affiliation (country)	Role
Betty Lucy López	Universidad de Antioquia (Colombia)	Panelist/guest expert
Filomena Freitas	Universidade Nova de Lisboa (Portugal)	Panelist/guest expert
Molly Morse	Mango Materials Company (USA)	Panelist/guest expert
Maria José Fabra	IATA-CSIC (Spain)	Panelist/guest expert
Başak Öztürk	Leibniz Institute DSMZ (Germany)	Panelist/guest expert
Tim Bugg	University of Warwick (UK)	Panelist/guest expert
Ren Wei	University of Greifswald (Germany)	Guest expert
Angela María Alvarado Fernández	Pontificia Universidad Javeriana (Colombia)	Panelist
Diego Javier Jiménez	Universidad de los Andes (Colombia)	Moderator/organizer
Felipe Salcedo Galán	Universidad de los Andes (Colombia)	Organizer
Juan Fernando Saldarriaga	Universidad de los Andes (Colombia)	Organizer
Jorge Medina	Universidad de los Andes (Colombia)	Guest expert
Carol Viviana Amaya Gomez	Agrosavia (Colombia)	Panelist/organizer
J. Lorena Castro Mayorga	Agrosavia (Colombia)	Organizer
Natalia A. Tarazona	Helmholtz-Zentrum Hereon (Germany)	Panelist/organizer
Hugo Pena-Cortes	Helmholtz-Zentrum Hereon (Germany)	Organizer
Judith Lehmann	Helmholtz-Zentrum Hereon (Germany)	Organizer
Rainhard Machatschek	Helmholtz-Zentrum Hereon (Germany)	Moderator/organizer
Andreas Lendlein	Helmholtz-Zentrum Hereon (Germany)	Organizer

**TABLE 3:** Research priorities in microbial biodegradation and recycling of plastics raised after the MENZYPOL-NET workshop 2021

Polymer science	Enzyme science	Microbial science
Unveiling the relationships between polymer chains structures and physicochemical properties of plastic materials and their biodegradability Use and standardization of modern techniques to accurately quantify plastic biodegradation at real-world conditions Development of novel biodegradable plastics synthesized by using plant and waste plastic-derived monomers Exploring strategies for more efficiently recycling/degrading both fossil-based and biobased plastics	Discovering the mode of action and natural substrates of PDE Engineering of novel PDE to enhance degradation and improve the economic feasibility of plastic biorecycling Design of novel strategies for directed enzyme evolution Exploring key enzymes and metabolic pathways involved in biodegradation of different types of plastics (fossil-based and biobased) Designing high-throughput screening assays directly addressing the plastic-degrading activity rather than activity on other model compounds	Exploring the evolution of PDE, PDM, and microbiomes impacted by (micro)plastics Understanding the impact of (micro)plastic input in environmental and host-associated microbiomes Isolation/recovery of novel PDM (bacteria and fungi) and design/characterization of PDC from different ecosystems Determining the actual degradative role of microbes within the plastisphere


## ARE DURABLE PLASTICS SUITABLE SUBSTRATES TO DRIVE MICROBIAL EVOLUTION?

Considering that mass production of plastic started in the 1950s, plastic littering is a very recent perturbation in the biosphere. This could be one of the reasons behind the still low efficiency of enzymatic-assisted of disassembly these polymers. However, microorganisms have adapted to metabolize other anthropogenic pollutants used in agriculture (such as pesticides), which have been found in nature since the 1960s (25). Microorganisms can evolve very quickly after a highly selective pressure; this happens due to their high genome plasticity, versatility, and capacity to exchange genetic material and to mix and match catabolic pathways. It is suggested that the strategies to degrade unknown pollutants are usually based on the evolution and adaptation of microbial enzymes that naturally act on other native substrates. Regarding the potential evolution of plastic-degrading microbes (PDM) and enzymes (PDE), there are still more open questions than answers. Are durable plastics (e.g., PET, PP, and PUs) suitable substrates to drive evolution? Could microbial life be at the very early stages of evolution when dealing with these polymers? Is it possible for microbes to have a fast adaptation to such fossil-based plastics in the coming decade?

Although there is so far no solid study that has verified the microbial and enzymatic biodegradation of durable plastics in nature, apparently there are fewer plastics in oceans than expected (the “missing plastic” paradox). This observation suggests that natural biodegradation processes could occur or that a substantial sink for plastic debris could be found in marine sediments (10, 26). Interestingly, there are some preliminary bioinformatic clues about the evolution and adaptation of putative PETases (i.e., PET hydrolases, enzymes that could hydrolyze PET) in global oceans (27). It has been reported that PET hydrolases occur at very low frequencies in marine and terrestrial metagenomes (28). Another recent metagenomic survey reported that ocean and soil microbiomes might already be adapting to current global plastic pollution trends (20). These conclusions (raised in the above-mentioned three studies) must be taken with caution because of the absence of biochemical analysis and because the native function of all identified/predicted PETases is still unknown. Moreover, plastic-degrading activities could be the promiscuity of certain enzyme classes that have their main activity on antique natural polymers (e.g., suberin and cutin, waxy compounds coating leaves) with chemical/physical features similar to those of plastics. However, the debate persists regarding whether microbial enzymes used to deconstruct natural plant polymers have had enough time to evolve and to adjust to equivalent functional groups present in durable plastics (26). In this sense, Dr. Öztürk pointed out that the natural evolution of environmental microorganisms to efficiently degrade plastic is likely to be very slow due to the recalcitrant nature of these polymers. In addition, Dr. Wei stated that as long as enough natural substrates (e.g., plant biomass) are available in the surrounding environment, there is no need for microbes to evolve to degrade plastics, as the energy required to break down the synthetic polymers should be markedly higher.

## RELEVANCE OF AND INTERPLAY BETWEEN PLASTICS, ENZYMES, AND ASSESSMENT METHODS

In-depth knowledge about the structural, physical, and chemical characteristics of polymeric materials (e.g., presence of hydrolyzable bonds along the chain, morphology, molecular weight, degree of crystallinity, glass transition temperature, and hydrophobicity) is highly relevant if efficient bioconversion systems (e.g., release and use of plastic monomers for recycling) are to be developed. These intrinsic properties will also define the enzymatic depolymerization mechanisms. The presence of additives, such as plasticizers and other impurities, should also be taken into account. These compounds could be easier to be broken down and catabolized by microbes than the plastic polymer itself, yielding false-positive results for plastic biodegradation (29, 30). The relevance of polymer chemistry in plastic biodegradation was also highlighted in a recent perspective article (31). As mentioned, microorganisms that could degrade plastics contain enzymes that work on natural substrates with structural similarities to them. In other words, if the microbes can by chance depolymerize plastics and catabolize their monomers, they do it through common known metabolic pathways used to degrade other carbon sources (32).

In light of this, would it still be possible to isolate efficient PDE from nature? To answer this question, it is highly relevant to understand (i) the “native” substrates of the enzymes with plastic-degrading capabilities, (ii) the kind of structures they can recognize, and (iii) the mechanisms of catalysis. Unfortunately, sometimes native substrates for enzymatic studies are not commercially available, and reliable enzymatic assays are difficult to set up. For example, in the case of cutinases, their activity has been tested using synthetic substrates like *p*-nitrophenol (*p*NP) esters and, in fewer cases, using cutin fibers (33, 34). Moreover, there is an urgent need to determine how to accurately evaluate the biodegradation of plastics. These methods can be focused on the changes in the physicochemical and mechanical properties after modification/disassembly of polymers (e.g., gravimetric measurements, Fourier transform infrared spectroscopy, atomic force microscopy, and/or thermogravimetric analysis), on the quantification of oligomer/monomer generation, on the metabolic products of microbial respiration (e.g., CO_2_), on the monitoring of oxygen consumption (e.g., using an OxiTop BOD system) (35), or a combination of the above (36). The detection of products after a biodegradation process can easily be performed (e.g., using high-performance liquid chromatography) for some hydrolyzable plastics, but in the case of polyolefins, specific and advanced techniques are required (37). Thus, we emphasize the importance of selecting and standardizing the techniques and methods that should be used to evaluate and accurately quantify the depolymerization of plastics, which would allow reproducibility between studies.

Other modern techniques, such as the Langmuir-Blodgett method, could also be adapted and used to evaluate physicochemical changes in plastics and to determine enzymatic kinetics in real-time measurements (38, 39). In the same way, the use of labeling techniques with stable isotopes could be an excellent option to trace the catabolism of plastics and CO_2_ production, identifying active microbes involved in this process, similar to what is reported for PBAT (an aromatic-aliphatic copolyester) biodegradation (40). Unfortunately, most of these labeled substrates are not yet commercially available. Finally, yet importantly, the development of bioinformatics tools and novel databases (e.g., PlasticDB, PMBD, and PAZy) (41–43) is crucial to identifying PDE from global metagenome/proteome data sets (20).

## BIODEGRADATION AND BIORECYCLING: KEY TOPICS IN THE CIRCULAR BIOECONOMY OF PLASTICS

Most plastics are products made for long-term use, and they are not made to be degraded; for instance, “we will not make PVC pipes biodegradable, because we need to replace them every couple of years,” said Dr. Öztürk. However, few durable plastics contain hydrolyzable bonds that could allow their biologically assisted depolymerization (biodegradation) into their respective oligomers/monomers (Table 1). Therefore, biodegradation of plastics could be partially achieved under lab-controlled and well-optimized conditions, which can be optimized for plastic recycling at an industrially related scale such as that demonstrated for PET (44). Although enzymatic degradation of other durable plastics such as PE, PP, and PS is so far not confirmed by reproducible research (29), these polymers are under intensive investigation to open new possibilities of biodegradation using engineered enzymes or microbes. As for the biodegradation of plastics in the environment, it is important to note that the end-of-life fate (e.g., landfills, composting facilities, or the open environment) and abiotic factors (e.g., temperature, pH, salinity, UV radiation, oxygen, and moisture) play important roles in the enzymatic-assisted depolymerization (13, 15). The biodegradability of plastics is determined by international standard methods (e.g., ISO 17556, ISO 14851, and ASTM D6991), which are different for industrial composting, home composting, soil, and aquatic ecosystems (13, 45). Thus far, scientific evidence has not supported the idea that durable plastics are biodegradable. When plastics are being enzymatically disassembled under laboratory conditions, they should not (yet) be categorized as fully biodegradable, until field studies under ISO conditions confirm this. For additional concepts and terminology about biodegradation of polymers, please refer to IUPAC recommendations (46).

In general terms, plastic recycling can be divided into four main classes: mechanical, chemical, thermolysis, and incineration (47, 48). Mechanical recycling refers to the use of plastic waste to produce secondary raw materials by applying physical processes, without (significantly) changing their chemical structure. All types of fossil-based plastics can be mechanically recycled. This is by far the most common technique. However, mainly due to the high costs involved in plastic collection and sorting, new biotechnological options (known as chemical recycling) are now being intensively explored. Chemical recycling can be divided into different categories (one of them is biorecycling), but it mainly deals with the recovery of useful plastic-derived oligomers/monomers, which will be reused to produce virgin polymers (recycling) or other high-value products (upcycling) (e.g., biobased plastics, biosurfactant and/or organic acids), offering end-of-life advantages (13, 48). Finally, biocatalytic recycling (or biorecycling) has shown potential for upscaling waste in refineries (especially with PET) (49, 50), but it is still very challenging with polyolefins or other hydrolyzable polymers, including PUs. In September 2021, Carbios (a French company) opened a demonstration plant to test the enzymatic recycling of PET. However, it is known that the economic feasibility at an industrial scale could be a bottleneck, mostly due to the mechanical preprocessing, the high cost of enzymes, and difficulties in product recovery. A comprehensive analysis of the process, technoeconomical viability, environmental and socioeconomical impact of the enzymatic recycling of PET was published by Singh et al. (51).

Recently, a promising strategy for the bio-upcycling of PET using a strain of Pseudomonas umsongensis that can catabolize PET-derived monomers to produce PHAs was proposed (52). Here, lab-adaptative evolution experiments, metabolic engineering, and flux balance analysis were essential to achieve those goals (50). More research in this direction is foreseen to achieve a shift to a sustainable plastic biorecycling industry. One promising strategy may be the use of plastic oligomers/monomers as microbial substrates (or carbon sources) for upcycling of plastic waste (53). In this regard, Dr. Jiménez stated (based on genomic clues obtained by his group) that Pseudomonas protegens strains could have a hidden potential to catabolize terephthalate to produce PHAs.

## ENGINEERING OF PLASTIC-DEGRADING ENZYMES

Frances Arnold (winner of the Nobel prize in chemistry, 2018) highlights in her Nobel Lecture (“Innovation by Evolution: Bringing New Chemistry to Life”) that there are a plethora of proteins out there just waiting to solve humankind’s problems, and directed evolution could be a versatile tool for developing biocatalysts for new environments and functional tasks. In this context, we would need to modify/adapt existing PDE to transform durable plastics more efficiently (comment from Dr. Tarazona), using protein engineering approaches such as directed evolution and rational design (54, 55). However, bigger efforts are needed to screen enzymes that show activity against the most durable plastics. In 2016, a PET-degrading enzyme was isolated from Ideonella sakaiensis (*Is*PETase), which showed relatively high activity under mesophilic conditions (56). In recent years of protein engineering, the efficiency of this enzyme has been improved considerably (more than 50-fold compared to the wild type in certain application scenarios) (57). The most successful strategy was to improve the thermostability of this enzyme, although the most thermostable variant of *Is*PETase so far is still less active in depolymerizing PET waste than other intrinsically thermophilic homologs (comment from Dr. Wei), for example, the thermostable leaf compost cutinase (LCC) found from a leaf-branch compost metagenome (33). Recently, Tournier et al. (44) reported the highest PET degradation rate to date (approximately 90% at pH 8.0 and 72°C for 10 h) using a variant of LCC. In addition, the authors showed a productivity of 16.7 g of terephthalate per L per h (200 g per kg of PET solid loading, with an enzyme concentration of 3 mg per g of PET). In this sense, the use of the LCC enzyme, as a prospective approach to PET recycling, holds great potential compared to any *Is*PETase mutant published so far. Therefore, different protein engineering approaches, benefiting from computational predictions for specific mutation hot spots based on known structure-function relationships, will be useful to further improve the enzymatic degradation efficiency to work in industrial settings (55, 57, 58).

## WHAT IS THE IMPACT OF (MICRO)PLASTICS ON ENVIRONMENTAL MICROBIOMES?

MPs and NPs are pollutants that could be harmful to many organisms (e.g., bacteria, zooplankton, animals and humans). They can be found in soils, aquatic ecosystems (e.g., rivers and oceans), wastewater treatment plants, air, and human blood (59). MPs and NPs are small enough to be taken up by many organisms, raising concerns about bioaccumulation and biomagnification (60). The negative impact of gut-internalized plastics and MPs in animal species is widely reported (61, 62). However, there is a lack of information on how the presence of MPs and NPs affects the structure, diversity, and functions of environmental microbiomes, especially in tropical soil ecosystems (stated Dr. Jiménez). The consequences of MP pollution in the microbiomes are (i) an increase of carbon sources and providing extra niches; (ii) toxicity caused by plastic additives (e.g., plastic leachates); (iii) microbial attachment to plastic surfaces, serving as a vector of microbial dispersal (e.g., pathogens and/or antibiotic-resistant genes); and (iv) an impact on biochemical cycles and increase in gene exchange (21, 63–65). A major problem with MPs is their high surface area/volume ratio and hydrophobicity, which allow them to absorb other harmful pollutants (e.g., plasticizers, persistent organic pollutants and heavy metals) and carry them into environments. However, the high surface area can be an advantage to improve microbial attachment and enzymatic attack (66). The use of plastic mulch films (e.g., PE or PLA) has been reported as the principal source of MPs in agricultural soil systems. Interestingly, MPs can also be formed by biodegradable plastics entering in the environment (67). The incorporation of them into the soil may affect the microbiome due to changes in gas exchange, increased temperature, and reduced light transmissivity. In addition, an input of MPs in soil could have eco-evolutionary implications and a direct impact on soil structure, water availability, enzymatic activities, and microbial biomass, diversity, and function (68–70).

Some physicochemical and biological methods have been proposed to remove and treat MPs from the environment (71). However, an efficient microbial or enzymatic technology to dispose of MPs still needs to be developed (72). From an applied microbial ecological perspective, it will be highly relevant to understand what would happen with different microbiomes facing MP disturbance. We can probably learn from microbial eco-enzymology (defined here as the study of enzymes and their role in microbial interactions and the modification of surrounding environments) and eco-evolution to improve biodegradation or biorecycling in industrial settings (73). Additionally, such knowledge would likely increase our ability to understand and predict the environmental consequences of plastic pollution based on the perturbation of microbiomes and their changes (21). From our point of view, the transformation of MPs and NPs by microbes is an area of research that needs to be intensified in coming years, in particular, to develop methods to accurately characterize and evaluate their biodegradation in field studies.

## PLASTIC-DEGRADING MICROBES AND WHERE TO FIND THEM

Based on the postulated principle in microbial ecology “everything is everywhere,” microorganisms with the potential to degrade plastics could be found in several environments, and one should not need to hunt for PDM in special places like the plastisphere. However, it is also proposed that the environment selects for better-adapted microorganisms (74, 75). It is then likely that PDM could be found more frequently in places with a high abundance of plastics or other chemical compounds with similar structures. With this premise, it was agreed that environments enriched with plastic residues (e.g., landfills, garbage dumps, and polluted soil/freshwater/ocean), plant polymers (e.g., forest soils), beeswax, long-chain hydrocarbons, oil, and other xenobiotics could be excellent places to isolate PDM. With the recent finding on the capability of different insect species (particularly the larvae of darkling beetles, wax moths, and meal moths) to consume and degrade different plastic polymers (76, 77), their gut microbiome has become a huge target for finding PDE. Similarly, it has been reported that cow rumen fluid can hydrolyze different synthetic aromatic polyesters, turning its associated microbial communities into a source of PDE (78). Moreover, it is known that higher temperatures can increase the flexibility of plastics, allowing accessibility to enzymatic attack. In this regard, thermophile microbiomes (e.g., hot springs or compost) could be a promising source of thermostable PDE (79). Other blue-carbon environments that are highly polluted with MPs or oil spills like mangrove soil and seagrasses are still underexplored and could contain novel PDM and PDE (e.g., α/β-hydrolases) (80, 81). In 2017, Auta et al. (82) recovered two isolates of *Bacillus* sp., from mangrove soils, that grew in a synthetic medium containing UV-treated MPs as the sole carbon source. Currently, the assessment of MPs input in mangrove soils is a topic of interest within the Mangrove Microbiome Initiative (83). Moreover, for the isolation and characterization of novel PDM from those environments, the design of novel high-throughput screening methods, including liquid-medium and agar plate assays with emulsified plastic nanoparticles, are still needed.

## CORRELATION BETWEEN PLASTIC BIODEGRADATION, LIGNOCELLULOSE, AND MICROBIAL CONSORTIA

Lignin is a naturally occurring polymer that is relatively inert to degradation, being attacked only by wood-rotting fungi and some soil bacteria that have developed enzymatic strategies for its depolymerization (84). These lignin-degrading enzymes (e.g., peroxidases, laccases, and monooxygenases) might be candidates for the rational design of PDE (stated Dr. Bugg) (26). Lignin contains C-C and C-O bonds susceptible to enzymatic cleavage. Similarly, C-C bonds and C-O bonds form the main backbone linkage of some durable plastics, including PP, PS, PE, and PET. Thus, it is proposed that enzymes acting on plant-derived compounds could transform different types of plastics (85). As we state above, microbiomes with a high abundance of plant polymers can be excellent places to hunt for PDM (78). In this sense, biodegradation of plastics could follow principles similar to those of plant biomass, such as the presence of specialists/generalists, enzymatic synergism, “division of labor,” or even cross-feeding events.

Comparable to lignocellulose, plastics could be seen as complex substrates that would require synergy between microbial populations to achieve their biodegradation. Recently, the degradation of PBAT by a marine microbial consortium showed that synergism and division of labor could be key mechanisms (86), similar to what is reported for lignin catabolism (87). Thus, the use of microcosms or enrichment liquid cultures could be excellent “top-down” strategies to select plastic-degrading microbial consortia (PDC) from environmental microbiomes (88). A proposed strategy to select PDC is the “dilution-to-stimulation” approach (89), where plastics (or their derived monomers) are used by the microbes as the sole carbon source. Here, supplementation with vitamins, trace elements, and Casamino Acids at the beginning of the enrichment cultures could increase the microbial growth and the possibility of cometabolizing the substrate, facilitating the selection of PDM. Recently, methods and protocols to set up the selection and cultivation of aerobic and anaerobic PDC were reported (90). Other strategies for the design of microbial consortia with plastic-degrading capacities have been proposed using “bottom-up” approaches (91, 92). There are still many challenges to developing, using, preserving, and commercializing these types of consortia, and more research is needed in this field (31). Fortunately, some international projects are tackling this topic right now (e.g., Enzycle, Enzyclic, BioICEP, and MIX-UP) (93).

## BIOBASED PLASTICS: ALTERNATIVES, CHALLENGES, AND PERSPECTIVES

Biodegradation and recycling could aid in solving the plastic crisis. However, the synthesis and use of biodegradable polymers (e.g., PLA, PHAs, and/or PBAT), for applications in which plastics could have a high risk of reaching the biosphere, could minimize the negative impact of fossil-based plastics in natural environments. Nonetheless, the rate of biodegradation of these polymers in the environment varies considerably depending on the ecosystem where they end up (45, 94), and biodegradation might not be achieved in the short periods estimated by their manufacturers. The biobased-plastic industry is an emerging solution to a transition toward a more sustainable and circular plastic economy (11). This is a fast-growth market, predicted to reach 2.62 MT by 2023 (95). However, biobased plastics represent only less than 1% of the total plastic production. Their inferior mechanical properties compared to traditional plastics and the difficulties in integrating bioplastic waste into existing recycling chains are the primary reasons for their current limited utilization (96).

A comprehensive review of the advantages and challenges in the transformation (i.e., synthesis, biodegradation, and recycling) of bioplastics in the context of an efficient circular economy was recently published (11). According to a relevant study (95), the most promising biobased polymers, based on market share value, are PLA, polyglycolic acid (PGA), PHAs, and biobased versions of fossil-based plastics (such as bio-PET and bio-PE). PHAs are biobased polymers that can be produced in a “tailored” manner, since their monomers are highly diverse, varying the final polymer properties (97). Although PHAs make up only a small percentage of the bioplastic market (~1.4%), their production is set to quadruple by 2024. According to Molly Morse (CEO and cofounder of Mango Materials Company), potential strategies such as the use of methanotrophic bacteria that convert methane to produce PHAs (specifically poly-3-hydroxybutyrate [PHB]) are emerging. The renewed interest in this topic is due to the abundance of methane and the implications of using a less expensive and nonfood feedstock (e.g., lignocellulose). Indeed, it has been calculated that the production of PHB from methane would use less energy than comparable products obtained from sugar (98). Furthermore, given the high degradability of PHAs and the effect of monomer composition on their biodegradation (39, 99), modeling the structure-degradation relationships in these polymers could reveal important insights into the degradable potential of structurally similar polymers.

Moreover, other biobased polymers such as starch are also promising alternatives for producing biodegradable plastic materials. Starch is the most common plant-based polysaccharide for the development of bioplastic films due to its cost-effectiveness, abundance, and film-forming properties (100). Thermoplastic starch (TPS) stands the highest in terms of production capacity, as these materials are already replacing plastics, particularly in the flexible film packaging sector (101). TPS is both biobased and biodegradable. However, high hydrophilicity and poor mechanical properties are the main drawbacks, which can be addressed by blending this material with other polymers, thus complicating the recycling and degradation after its end of life (102). Composite blends of different fossil-based polymers with other natural additives, such as starch, lignin, or other natural fibers, are continuously being developed. They have shown potential for major uses in sustainable packaging, with the promise of being able to expand to a wide range of potential industrial applications (103). However, special care and characterization must be performed, since blending could also reduce the biodegradability of polymers in specific end-of-life environments (45). Furthermore, as briefly mentioned, it has been suggested that the synthesis of biobased polymers could aid the upcycling of plastics, by using the plastic-derived monomers as carbon sources for bioplastic-producing microorganisms, for instance, using engineered PETases to release terephthalate (TPA) and ethylene glycol (EG) monomers, which are later transformed by recombinant bacteria encoding the enzymes for the biosynthesis of PHAs (50), or cocultivating two microbes, one that is responsible for the degradation of PET to TPA and EG and the one that is responsible for the synthesis of PHB (104). These strategies could be considerably more efficient and must be improved in future research.
